# Association of insulin resistance with visual decline in older individuals without diabetes: a cross-sectional mediation analysis

**DOI:** 10.3389/fendo.2026.1758444

**Published:** 2026-03-05

**Authors:** Tomoyuki Matsuyama, Hanako Nakajima, Hiroshi Okada, Koji Kitazawa, Yohei Yamashita, Naoko Nakanishi, Masahide Hamaguchi, Satoaki Matoba, Chie Sotozono, Michiaki Fukui

**Affiliations:** 1 Department of Endocrinology and Metabolism, Graduate School of Medical Science, Kyoto Prefectural University of Medicine, Kyoto, Japan; 2 Department of Ophthalmology, Kyoto Prefectural University of Medicine, Kyoto, Japan; 3 Department of Cardiovascular Medicine, Kyoto Prefectural University of Medicine, Kyoto, Japan

**Keywords:** body composition, individuals without diabetes, insulin resistance, older individuals, visual impairment

## Abstract

**Aims:**

It remains unknown how visual decline affects insulin resistance in individuals without diabetes. Therefore, we investigated the association between visual impairment and insulin resistance in older individuals without diabetes.

**Methods:**

Data from the Kyotango Longevity Cohort Study, a prospective investigation encompassing adults aged 65 years and above, were included. The study protocol was approved by the Ethics Committee of the Kyoto Prefectural University of Medicine (ERB-C-885). Visual acuity and insulin resistance were assessed using logarithmic minimum angle of resolution (logMAR) and homeostasis model assessment ratio (HOMA-R), respectively. The correlation between logMAR and HOMA-R was examined using Spearman’s rank correlation coefficient and multiple regression analysis. Causal mediation analysis was performed with body composition as a mediating factor.

**Results:**

In total, 797 participants were enrolled. Mean age was 74.8 ± 6.6 years in men and 73.4 ± 5.6 in women. logMAR and HOMA-R were correlated in women without diabetes after adjustment for covariates (β = 0.166, P = 0.0002). Causal mediation analysis revealed that body fat mass (proportion mediated: 0.567, 95% CI: 0.058–0.938) and body fat percentage (proportion mediated: 0.685, 95% CI: 0.250–1.027) significantly mediated the relationship between logMAR and HOMA-R in women.

**Conclusions:**

Vision decline was associated with insulin resistance, with body fat statistically accounting for part of this association.

## 1 Introduction

In Japan, improvements in the living environment and advances in medical science have increased the average life expectancy, and it is among the countries with the longest life expectancy worldwide. However, Japan is facing a rapidly aging population and an increase in lifestyle-related diseases, such as diabetes. These trends have emerged as major social problems, leading to a higher number of people requiring nursing care and increasing pressure on medical costs.

Diabetes mellitus, characterized by chronic hyperglycemia caused by inadequate insulin action, results in various complications. Although various causes of diabetes are known, increased insulin resistance due to decreased muscle mass, increased fat mass caused by physical inactivity, and poor dietary habits are important factors in the development of lifestyle-related diseases, including diabetes (1), hypertension, and hyperlipidemia (2). Prolonged hyperglycemia caused by insulin resistance leads to metabolic abnormalities. These can result in macrovascular and microvascular disorders, such as coronary artery disease, stroke, diabetic retinopathy, nephropathy, and neuropathy, which significantly threaten quality of life and longevity (3).

The incidence of various eye diseases increases with age, and visual function eventually declines (4–7). Older adults with visual impairments often refrain from going out, resulting in reduced physical activity and a progressive decline in motor function, which may eventually lead to being bedridden. Impaired vision compromises mobility and increases the risk of falls and fractures (8–10). Thus, visual decline in older adults is a major public health concern that affects their daily lives and functional independence.

Although diabetes can lead to visual decline through diabetic retinopathy (11), it is unknown how visual decline affects insulin resistance in individuals without diabetes. This relationship is important because visual impairment may restrict physical activity. Such restrictions can potentially contribute to insulin resistance by affecting body composition, including muscle and fat mass, even in individuals without diabetes. Therefore, we investigated the association between visual decline and insulin resistance and performed a causal mediation analysis with body composition as a mediating factor in older individuals without diabetes.

## 2 Methods

### 2.1 Study design and participants

We included data from the Kyotango Longevity Cohort Study, a prospective investigation of adults aged 65 years and older residing in Kyotango City, a rural area in northern Kyoto Prefecture, Japan. This was a long-term longitudinal project, spanning August 2017 to March 2050, aimed at identifying factors contributing to healthy longevity. The cohort study was conducted in collaboration with the Kyoto Prefectural University of Medicine, local public health authorities, and 13 universities nationwide participating in the “Center of Innovation (COI) Project led by Hirosaki University. All eligible participants meeting the inclusion criteria were included in the present analysis. The source population comprised all adults aged ≥65 years who participated in the Kyotango Longevity Cohort Study, and the study population consisted of eligible participants included in the present analysis after applying the predefined exclusion criteria. Participants with diabetes and those with missing data were excluded. Participants with hypertensive or arteriosclerotic changes in fundus examination were excluded. As this study was a secondary analysis of an existing population-based cohort, a formal sample size calculation was not performed. The study protocol was approved by the Ethics Committee of the Kyoto Prefectural University of Medicine (ERB-C-885, approved on July 20, 2017). All participants provided written informed consent before enrollment, and the study was conducted in accordance with the tenets outlined in the Declaration of Helsinki.

### 2.2 Definition of diabetes

Diabetes was defined as a fasting blood glucose level of 126 mg/dL or higher and HbA1c of 6.5% or higher, those undergoing diabetes treatment, or with a history of diabetes.

### 2.3 Data collection

Fasting blood tests were performed during cohort enrolment. Data on medication use, smoking status, alcohol consumption, and educational level were collected using a standardized questionnaire. Participants were classified as current, past, or never smokers; those who consumed more than 20 g of ethanol per day were defined as drinkers; and those who had attended university, junior college, or vocational school were considered to have an academic background.

Fasting blood insulin level and homeostasis model assessment ratio (HOMA-R) are simple indicators of insulin resistance under conditions where insulin secretion is maintained (12). HOMA-R was calculated as: HOMA-R = fasting blood glucose level (mg/dL) × fasting insulin level (μU/mL)/405 (13). Fibrosis 4 (FIB-4) index, an indicator of liver fibrosis resulting from insulin resistance, was calculated as: FIB-4 index = age × aspartate aminotransferase (IU/L) /platelet count (109/L) × √alanine aminotransferase (IU/L) (14).

### 2.4 Visual assessment and fundus examination

Visual acuity assessments and fundus examinations were performed by professional orthoptists. The best-corrected visual acuity was measured using a standard Japanese visual acuity chart. Visual acuity was assessed using the logarithmic minimum angle of resolution (logMAR) scale. This scale applies the logarithm of the visual angle to ensure equal spacing between acuity levels. LogMAR values were calculated from decimal visual acuity using the following formula: logMAR = - log_10_ (decimal visual acuity) (15).

### 2.5 Body composition

Body mass index (BMI) (kg/m^2^) was calculated as weight (kg) divided by height squared (m^2^). Body composition was assessed using a multifrequency bioelectrical impedance analyzer (InBody 770, InBody Japan, Tokyo, Japan). This included fat mass, appendicular skeletal muscle mass, soft lean mass, and fat-free mass. Skeletal muscle mass index (SMI) was calculated as: SMI (kg/m²) = appendicular skeletal muscle mass (kg)/height (m²). The body fat percentage was calculated as: body fat percentage (%) = (fat mass (kg)/body weight (kg)) × 100.

### 2.6 Statistical analysis

Continuous variables are presented as mean ± standard deviation (SD) for normally distributed data and as median (interquartile range) for non-normally distributed data. Categorical variables are expressed as numbers and percentages. All analyses were conducted separately for both sexes. First, the correlation between logMAR and HOMA-R was examined using Spearman’s rank correlation coefficient and multiple regression analysis after adjusting for age, BMI, smoking status (current or past smoker), alcohol consumption, education, and FIB-4 index (Model 1). The same analysis was performed to determine the association between logMAR and immunoreactive insulin (IRI).

**Figure 1:**
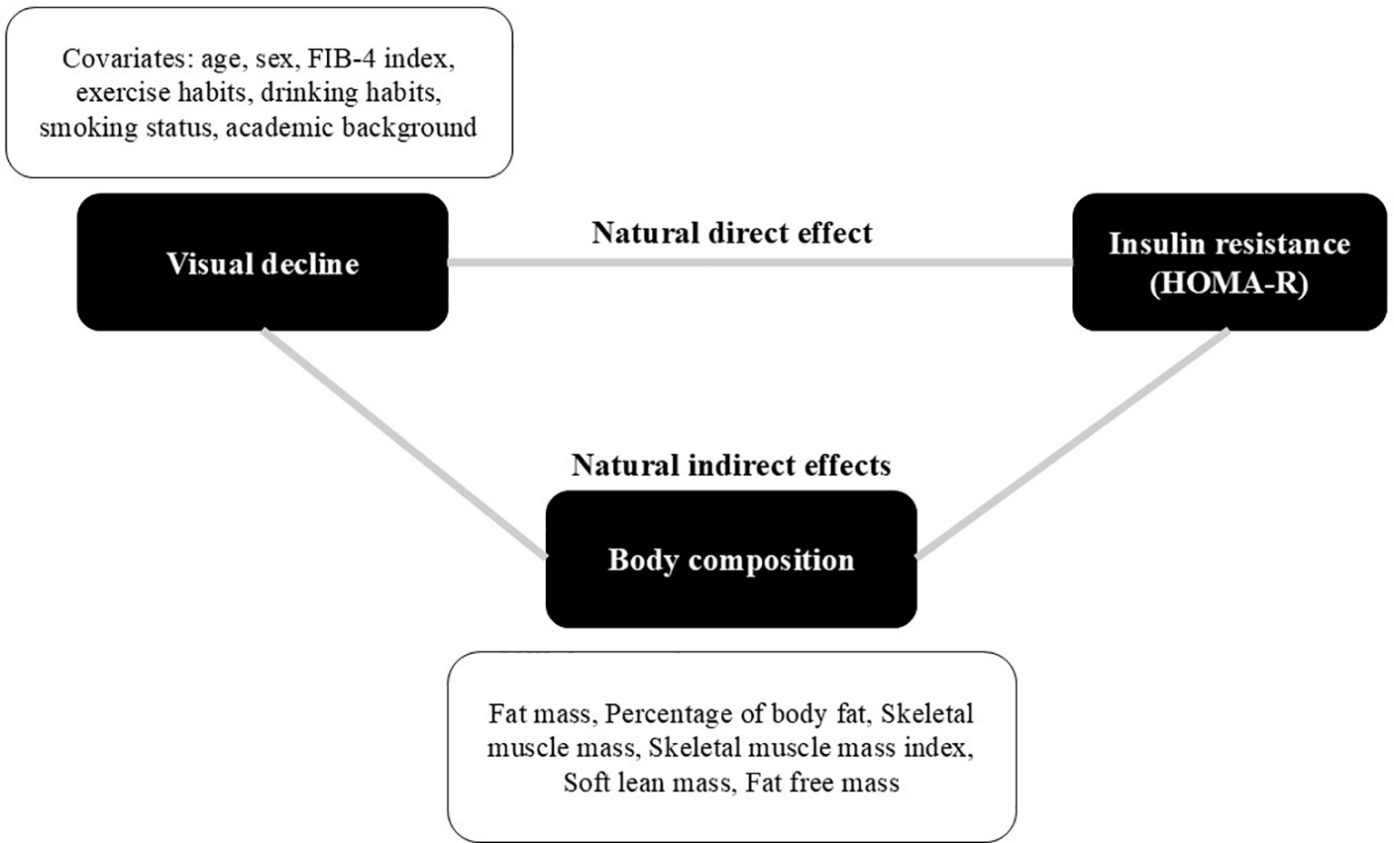
A concept of causal mediation analysis.

The estimated ‘total-effect’ regression coefficient was decomposed by causal mediation analysis. Body composition (i.e., fat mass, body fat percentage, skeletal muscle mass, SMI, soft lean mass, and fat-free mass) was considered as a single mediator in the analysis. Conceptualized relationships between visual acuity, body composition, and HOMA-R are shown in **Figure 1**. The total-effect regression coefficient was decomposed into a natural indirect effect by supposed body composition and a natural direct effect. The mediation proportion was calculated as the indirect effect divided by the total effect.

The aforementioned variables were adjusted for as confounders in the mediation model, except for BMI. An interaction term between exposure (logMAR) and the mediator (body composition) was included in the model. In the causal mediation analysis, 95% confidence intervals (CIs) were estimated by repeating the analysis with 2000 bootstrap samples.

As a subgroup analysis, we excluded participants with major eye diseases that may substantially affect visual acuity and are potentially associated with obesity, including untreated cataract, glaucoma, age-related macular degeneration, and retinal vein occlusion. Information on eye diseases was obtained using a self-administered questionnaire.

Several sensitivity analyses were performed. First, in addition to the primary adjustment model (Model 1: age, BMI, FIB-4 index, smoking status, alcohol consumption, and education), an extended model (Model 2) further adjusted for testosterone, estradiol, and sleep duration to account for potential hormonal and circadian influences. Second, both the main and subgroup analyses were repeated using best-corrected visual acuity instead of logMAR as the indicator of visual function. Third, standardized indirect effects were calculated to assess the robustness of mediation findings to scale dependence.

Statistical analyses were performed using JMP Pro version 18.1.0 software (SAS Institute Inc., Cary, North Carolina, USA) and R version 4.4.2 software (R Development Core Team). P<0.05 was considered statistically significant. The “CMAverse” package in R software was used to estimate the direct and indirect effects on the regression coefficient (16).

## 3 Results

### 3.1 Participant selection flowchart and baseline characteristics

**Figure 2:**
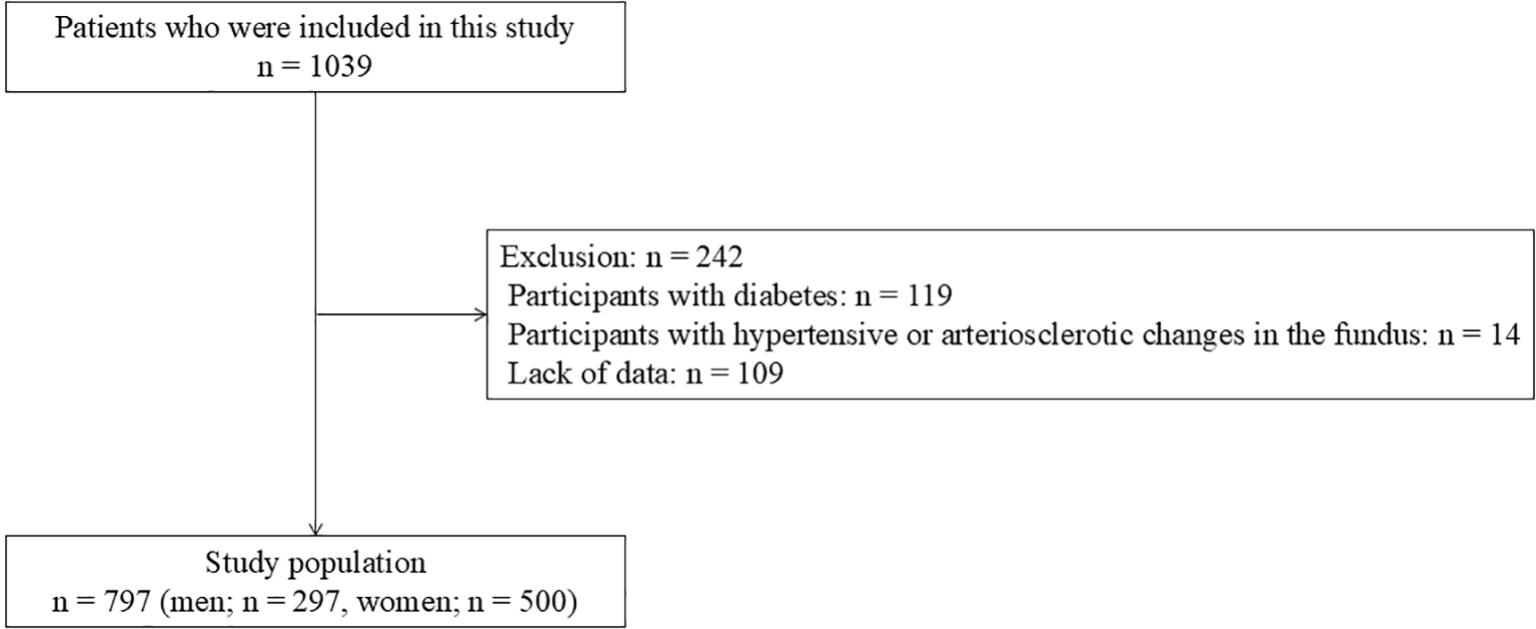
Flowchart of study participant selection.

**Figure 2** shows the flowchart of study participant selection. This study included 1,039 patients in the Kyotango Longevity Cohort Study from 2017 to 2023. Of these, participants with diabetes (n = 119), hypertensive changes in the fundus (n = 14), or missing data (n = 109) were excluded. A total of 797 participants were included in this study.

**Table 1** shows the clinical characteristics of the participants in this study. Of the 797 participants, 297 were men (mean age: 74.8 ± 6.6 years, BMI: 23.4 ± 3.0 kg/m^2^) and 500 were women (mean age: 73.4 ± 5.6 years, BMI: 22.9 ± 3.2 kg/m^2^). The median HOMA-R and logMAR were 1.1 (0.8–1.7) and 0.0 (-0.079 to 0.023) for men and 1.2 (0.8–1.7) and -0.079 (-0.079 to 0.0) for women, respectively. The distribution of logMAR by sex is shown in the **Supplementary Figure 1**.

**Table 1:** Characteristics of study participants.

Clinical characteristics	Total (n=797)	Men (n=297)	Women (n=500)
Sex (%)	797 (100)	297 (37)	500 (63)
Age (years)	73.9 ± 6.0	74.8 ± 6.6	73.4 ± 5.6
Body mass index (kg/m^2^)	23.1 ± 3.1	23.4 ± 3.0	22.9 ± 3.2
Fat mass (kg) (n=794)	16.5 ± 5.6	15.6 ± 5.5	17.0 ± 5.6
Percentage of body fat (%) (n=794)	29.0 ± 7.5	24.3 ± 6.3	31.7 ± 6.8
Skeletal muscle mass (kg) (n=794)	21.3 ± 4.5	25.9 ± 3.6	18.6 ± 2.2
Skeletal muscle mass index (kg/m^2^) (n=794)	6.4 ± 1.0	7.3 ± 0.7	5.9 ± 0.6
Soft lean mass (kg) (n=794)	37.5 ± 7.1	44.8 ± 5.7	33.3 ± 3.5
Fat free mass (kg) (n=794)	39.8 ± 7.5	47.3 ± 6.0	35.3 ± 3.7
Systolic blood pressure (mmHg)	138.0 ± 17.6	137.7 ± 18.4	138.1 ± 17.2
Diastolic blood pressure (mmHg)	76.6 ± 10.0	77.5 ± 10.4	76.1 ± 9.7
TG (mg/dL)	115.8 ± 62.2	120.2 ± 62.8	113.2 ± 61.8
HDL-C (mg/dL)	68.9 ± 17.7	63.5 ± 16.9	72.1 ± 17.4
LDL-C (mg/dL)	123.5 ± 30.9	118.9 ± 28.7	126.3 ± 31.8
FIB-4 index	2.0 ± 0.7	2.1 ± 0.8	1.9 ± 0.7
HbA1c (%)	5.7 ± 0.4	5.7 ± 0.4	5.7 ± 0.4
Fasting plasma glucose (mg/dL)	101.4 ± 11.5	102.2 ± 11.2	100.9 ± 11.6
IRI (μU/mL)	4.6 (3.4-6.7)	4.4 (3.2-6.5)	4.8 (3.6-6.9)
HOMA-R	1.2 (0.8-1.7)	1.1 (0.8-1.7)	1.2 (0.8-1.7)
Free testosterone (pg/mL)	2.2 ± 2.9	5.5 ± 2.1	0.2 ± 0.2
Estradiol (pg/mL) (n=796)	13.2 ± 10.8	23.1 ± 9.4	7.3 ± 6.4
BCVA	1.11 ± 0.26	1.11 ± 0.28	1.11 ± 0.25
LogMAR	-0.079 (-0.079-0.0)	0.0 (-0.079-0.023)	-0.079 (-0.079-0.0)
Current smoker (yes) (%)	35 (4.4)	29 (3.6)	6 (0.8)
Past smoker (yes) (%)	153 (19.2)	144 (18.1)	9 (1.1)
Non-smoker (yes) (%)	609 (76.4)	124 (15.6)	485 (60.9)
Drinking (yes) (%)	304 (38)	199 (67)	105 (21)
Academic background (%)	223 (28)	94 (32)	129 (26)
Sleep duration (hours) (n=773)	7.2 ± 1.1	7.4 ± 1.2	7.0 ± 1.1

TG, Triglyceride; HDL-C, High Density Lipoprotein Cholesterol; LDL-C, Low Density Lipoprotein Cholesterol; FIB-4 index, Fibrosis 4 index; IRI, Immunoreactive insulin; HOMA-R, Homeostasis model assessment ratio; BCVA, Best-corrected visual acuity; LogMAR, Logarithmic minimum angle of resolution.

### 3.2 Primary analysis

**Table 2** shows the relationships between logMAR and HOMA-R and IRI. After adjusting for confounders using multiple regression analysis, logMAR was significantly correlated with HOMA-R in women (β = 0.166, p = 0.0002) but not in men (β = -0.015, p = 0.7986). Similarly, logMAR was significantly correlated with IRI in women. When the interaction between logMAR and HOMA-R by sex was examined, the interaction term was significant (p = 0.0032), indicating that the association between logMAR and HOMA-R differed by sex.

**Table 2:** Relationship between logMAR and HOMA-R.

HOMA-R	Men (n=297)		Women (n=500)		Interaction
Regression analysis	β	p	β	p	p
Simple regression analysis	0.010	0.8591	0.233	<0.0001	0.0002
Multiple regression analysis (Model 1)	-0.015	0.7986	0.166	0.0002	0.0032
Multiple regression analysis (Model 2)	-0.015	0.8068	0.157	0.0006	0.0027
(Men/Women (n=287/485))					
IRI	Men (n=297)		Women (n=500)		Interaction
	β	p	β	p	p
Simple regression analysis	0.013	0.8222	0.23	<0.0001	0.0003
Multiple regression analysis (Model 1)	0.0003	0.995	0.161	0.0002	0.0077
Multiple regression analysis (Model 2)	0.001	0.9841	0.152	0.0006	0.0058
(Men/Women (n=287/485))					

Model 1 was adjusted for age, body mass index (BMI), FIB-4 index, smoking status (current or past smoker), drinking habits, and academic background.

Model 2 was further adjusted for free testosterone, estradiol, and sleep duration.

LogMAR, Logarithmic minimum angle of resolution; HOMA-R, Homeostasis model assessment ratio; IRI, Immunoreactive insulin.The sample size indicated as (Men/Women (n=287/485)) refers to Multiple regression analysis (Model 2).

### 3.3 Causal mediation analysis

**Table 3** shows the results of the causal mediation analysis of the relationship between logMAR and HOMA-R in women. In the primary adjustment model (Model 1), causal mediation analysis revealed that body fat mass and body fat percentage mediated the relationship between logMAR and HOMA-R. The proportions mediated by body fat mass and percentage of body fat were 0.567 (95% CI: 0.058–0.938) and 0.685 (95% CI: 0.250–1.027), respectively. In contrast, causal mediation analysis using skeletal muscle mass, SMI, soft lean mass, and fat-free mass did not reveal any significant indirect effects. Similar mediation proportions were observed in Model 2.

**Table 3:** Total, direct, and indirect effects of logMAR on HOMA-R in various mediating factors.

Exposure	Mediator	Total effect	p	Natural direct effect	p	Natural indirect effect	p	Proportion mediated (%)	p
LogMAR	Fat mass Model 1	6.152 (0.830-12.129)	0.011	5.604 (0.667-11.158)	0.014	3.487 (0.095-8.609)	0.041	0.567 (0.058-0.938)	0.042
	Model 2	5.522 (0.503-12.356)	0.020	4.983 (0.292-11.359)	0.027	3.370 (0.015-8.811)	0.047	0.610 (0.004-0.996)	0.049
	Percentage of body fat Model 1 Model 2	6.530 (1.109-13.896) 6.701 (0.661-13.743)	0.006 0.014	5.912 (0.818-12.749) 6.074 (0.335-12.589)	0.012 0.026	4.471 (0.428-10.890) 4.473 (0.209-10.785)	0.018 0.023	0.685 (0.250-1.027) 0.667 (0.218-1.185)	0.020 0.025
	Skeletal muscle mass Model 1 Model 2	3.664 (0.177-8.156) 3.381 (0.032-8.053)	0.034 0.050	3.787 (0.359-8.237) 3.530 (0.202-8.110)	0.019 0.038	-0.119 (-1.396-1.785) -0.200 (-1.578-2.184)	0.836 0.793	- -	0.868 0.839
	Skeletal muscle mass index Model 1 Model 2	3.880 (0.571-8.653) 3.528 (0.361-7.668)	0.011 0.014	3.812 (0.664-8.335) 3.483 (0.411-7.450)	0.008 0.013	0.351 (-1.156-2.948) 0.241 (-1.337-2.515)	0.690 0.769	- -	0.685 0.759
	Soft lean mass Model 1 Model 2	3.703 (0.383-8.344) 3.430 (0.031-8.284)	0.025 0.047	3.815 (0.545-8.350) 3.568 (0.248-8.338)	0.017 0.033	-0.068 (-1.255-2.040) -0.136 (-1.543-2.249)	0.912 0.891	- -	0.937 0.932
	Fat free mass Model 1 Model 2	3.752 (0.316-8.066) 3.477 (0.052-8.373)	0.027 0.047	3.865 (0.495-8.132) 3.615 (0.269-8.386)	0.019 0.031	-0.018 (-1.302-2.199) -0.080 (-1.590-2.526)	0.941 0.926	- -	0.966 0.969

Model 1 was adjusted for age, FIB-4 index, smoking status (current or past smoker), drinking habits, and academic background.

Model 2 was further adjusted for free testosterone, estradiol, and sleep duration.

LogMAR, Logarithmic minimum angle of resolution; HOMA-R, Homeostasis model assessment ratio.

### 3.4 Sensitivity and subgroup analyses

In the overall population, an additional analysis using best-corrected visual acuity as an alternative measure of visual function showed results largely consistent with those of the primary analysis, with a significant association observed between best-corrected visual acuity and HOMA-R (**Supplementary Table 2A**). In the corresponding causal mediation analysis, body fat percentage, but not absolute fat mass, showed a statistically significant mediating effect on the association between best-corrected visual acuity and HOMA-R (**Supplementary Table 3A**).

The flowchart of participant selection in the subgroup analysis is shown in **Supplementary Figure 2**, and the characteristics of study participants are presented in **Supplementary Table 1**. In this sub-analysis excluding participants with major eye diseases, including untreated cataract, glaucoma, age-related macular degeneration, and retinal vein occlusion, the associations between logMAR visual acuity and HOMA-R, as well as between best-corrected visual acuity and HOMA-R, were largely consistent with those observed in the primary analysis (**Supplementary Tables 2B, C**). In these sub-analyses, causal mediation analyses showed that neither fat mass nor body fat percentage exhibited a statistically significant mediating effect (**Supplementary Table 3B, C**).

To further improve interpretability, standardized indirect effects were calculated. In the overall population, standardized indirect effects were significant for both fat mass and percentage of body fat in the association between logMAR and HOMA-R, whereas neither showed statistically significant effects in the association between BCVA and HOMA-R (**Supplementary Table 4A**). In contrast, in the subgroup analysis excluding participants with eye diseases, standardized indirect effects for both fat mass and percentage of body fat were not statistically significant in either association (**Supplementary Table 4B**).

## 4 Discussion

In this study, the association between visual decline and insulin resistance in individuals without diabetes was examined. When the results were analyzed separately by sex, no significant association was observed in men, whereas a significant association was observed in women. In women, body fat mass and body fat percentage statistically accounted for part of the association between logMAR and HOMA-R.

We considered both the direct and indirect factors in the relationship between visual decline and insulin resistance. Biological rhythms are importantly involved in this relationship. Biological rhythms, represented by sleep and deep body temperature, are involved in many physiological phenomena, such as endocrinology, metabolism, circulation, and mental functions. Epidemiological studies have shown that the disruption of these biological rhythms is associated with various diseases, including obesity, dyslipidemia (17), diabetes (18), hypertension (19), sleep disorders, depression (20), stroke, ischemic heart disease (21), and cancer (22).

Photoreception in the retina is important for maintaining biological rhythms and adaptation to the external environment. Light stimulation of the retina is through the light-sensitive retinal ganglion cells and is transmitted to the suprachiasmatic nucleus, the center of the biological rhythm, to synchronize the biological rhythm of the peripheral tissues (23). In patients with cataract, lens opacity reduces photoreception in the retina, likely leading to disturbances in the biological rhythm. With aging, the lens tends to block short-wavelength light that excites light-sensitive retinal ganglion cells (24). Therefore, in older individuals, visual impairment may be associated with disturbances in biological rhythms, and these factors may be associated with insulin resistance.

However, possible indirect pathways linking visual decline and insulin resistance may involve diet and physical activity. Although data on diet and physical activity were not available in the present study, we hypothesized that visual decline is associated with diet and physical activity patterns, and that these factors are associated with greater body fat mass, which in turn is associated with insulin resistance. In a study examining the dietary habits of visually impaired individuals aged 25–74 years, obese participants were reported to consume fewer green and yellow vegetables compared with non-obese individuals (25). In a study of Indian older adults aged 65 to 83 years, although the difference was not statistically significant, visually impaired individuals tended to consume vegetables less frequently than those with normal vision (26). Additionally, higher vegetable intake was associated with a lower tendency toward obesity (27). Visually impaired individuals may spend less time and effort on cooking due to the burden and anxiety of using fire, leading to reduced vegetable intake. Studies examining physical activity among visually impaired adults have reported that visually impaired individuals have less physical activity, including walking and moderate-to-vigorous physical activity (MVPA) (28, 29). Visually impaired individuals are less physically active than non-visually impaired individuals, and lower physical activity is associated with the risk of lifestyle-related diseases (30–33). Visually impaired individuals often limit their physical activity because of fear of falling and perceived risk of mobility (34–36).

As discussed above, visual decline may be associated with greater body fat and insulin resistance, potentially in relation to biological rhythm disturbances (direct factors) and diet and physical activity patterns (indirect factors). In the present study, mediation analysis suggested that body fat statistically accounted for part of the association between visual decline and insulin resistance.

Sex differences in the association between vision and insulin resistance may be influenced by differences in sociobehavioral factors, such as housework. A study aimed at clarifying the actual conditions of the frequency and duration of MVPA using activity meter data among community-dwelling older adults found that, while there were no significant sex differences in total MVPA duration, women performed short bouts of MVPA lasting 1–4 minutes more frequently than men (37). Previous studies on physical activities have generally shown that men are more active regardless of age (38), but this is not necessarily the case when focusing on short bouts of MVPA (37). The greater frequency of short bouts of MVPA among women may be due to lifestyle differences between Japanese men and women. Even in modern times, Japanese women perform more household chores than men (39). They also engage in more daily activities, such as cleaning and laundry, which may be captured as short bouts of MVPA (37). These daily activities are more likely to be affected by visual impairment, and women who are responsible for more household chores may be more affected by visual impairment, which may be a factor in the observed sex differences.

In the present study, the association between visual function and insulin resistance was observed only in women, even after additional adjustment for sex hormones and sleep duration. Specifically, while Model 1 adjusted for age, BMI, FIB-4 index, smoking and drinking status, and educational background, Model 2 further included testosterone, estradiol, and sleep duration, and the association between logMAR and HOMA-R in women remained essentially unchanged. These findings suggest that the observed sex-specific association cannot be fully explained by differences in circulating sex hormones or sleep duration alone. In addition to sociobehavioral factors, biological differences between women and men in older adults—including sex differences in overall adiposity, with women generally having greater total fat mass; differences in fat distribution, whereby men tend to accumulate more visceral fat and women more subcutaneous fat (40); sex-specific metabolic characteristics; and retinal physiological differences, as estrogen receptors are present in retinal tissues and declining estrogen levels, particularly in postmenopausal women, may influence visual function and susceptibility to retinal diseases (41)—may affect the detectability of the association between visual function and insulin resistance.

In the present mediation analyses, relatively large mediation proportions with wide confidence intervals, including values exceeding 100%, were observed. Such estimates can arise in causal mediation analysis when the total effect is small or when the direct and indirect effects have opposite signs, and they do not necessarily indicate complete or excessive mediation. Accordingly, these mediation proportions should be interpreted with caution and should not be regarded as evidence of definitive causal mechanisms. To improve interpretability and address scale dependence, we additionally calculated standardized indirect effects, which are less sensitive to measurement units and facilitate comparison across models. These standardized estimates are presented in the Supplementary Tables and are intended to provide complementary, exploratory information rather than confirmatory evidence of mediation.

In this sub-analysis, no significant mediating effects of fat mass or percentage of body fat were observed in the relationship between logMAR and HOMAR. This may be partly due to the exclusion of eye diseases causing visual impairment, which led to the exclusion of some participants with visual decline and, consequently, smaller potential mediating effects of fat mass and percentage of body fat.

This study has several limitations that warrant consideration. First, the cross-sectional design limits the ability to infer temporal ordering or causal relationships between visual function and insulin resistance. Although an association between visual decline and insulin resistance was observed, reverse causality—whereby insulin resistance in individuals without diabetes may also affect visual function—cannot be excluded. Second, although we adjusted for several potential confounders, including age, BMI, FIB-4 index, smoking and drinking status, and educational background, detailed information on dietary habits and physical activity was not available and therefore could not be included in the analysis. As a result, residual confounding due to unmeasured lifestyle factors cannot be ruled out. In addition, information on eye diseases was obtained through a self-administered questionnaire rather than clinical verification, which may have introduced misclassification bias. Furthermore, interpretations regarding sex-specific differences were based on external evidence and sociobehavioral assumptions rather than direct measurements within the present cohort, which may limit their validity and generalizability. Third, the study population consisted of older Japanese individuals residing in rural areas. Therefore, caution is warranted when generalizing the present findings to younger populations, urban residents, or individuals from different ethnic or cultural backgrounds. Furthermore, mediation analysis relies on strong assumptions, including correct temporal ordering and the absence of unmeasured confounding, which cannot be fully verified in a cross-sectional setting. In addition, mediation proportions were sensitive to the scale properties of visual function measures, and therefore should be interpreted cautiously. Accordingly, the observed mediation effects should not be interpreted as evidence of definitive causal mechanisms, but rather as exploratory and hypothesis-generating findings. Future longitudinal studies are required to more accurately examine the association between visual impairment and insulin resistance in individuals without diabetes and to clarify the directionality of this relationship.

## 5 Conclusion

In the present study, logMAR and HOMA-R were significantly correlated in women without diabetes. These findings suggest that visual impairment may be associated with a higher level of insulin resistance. In addition, visual function may warrant consideration in future metabolic research, and preventive medicine involving older adults without diabetes. However, as this study was cross-sectional, the possibility that insulin resistance may also affect visual function cannot be ruled out. Future longitudinal studies are needed to more accurately examine the association between visual impairment and insulin resistance in individuals without diabetes and to clarify the directionality of this relationship.

## Data Availability

The raw data supporting the conclusions of this article will be made available by the authors, without undue reservation.

## Supplementary material

Supplementary Figure 1 shows the distribution of logMAR by sex.

Supplementary Figure 2 shows the flowchart of participant selection for the subgroup analysis.
