# Supplementary figures and images for: Association of insulin resistance with visual decline in older individuals without diabetes: a cross-sectional mediation analysis
Source: Front Endocrinol (Lausanne). 2026 Mar 5;17:1758444. doi: 10.3389/fendo.2026.1758444 (PMC12999418; doi:10.3389/fendo.2026.1758444)

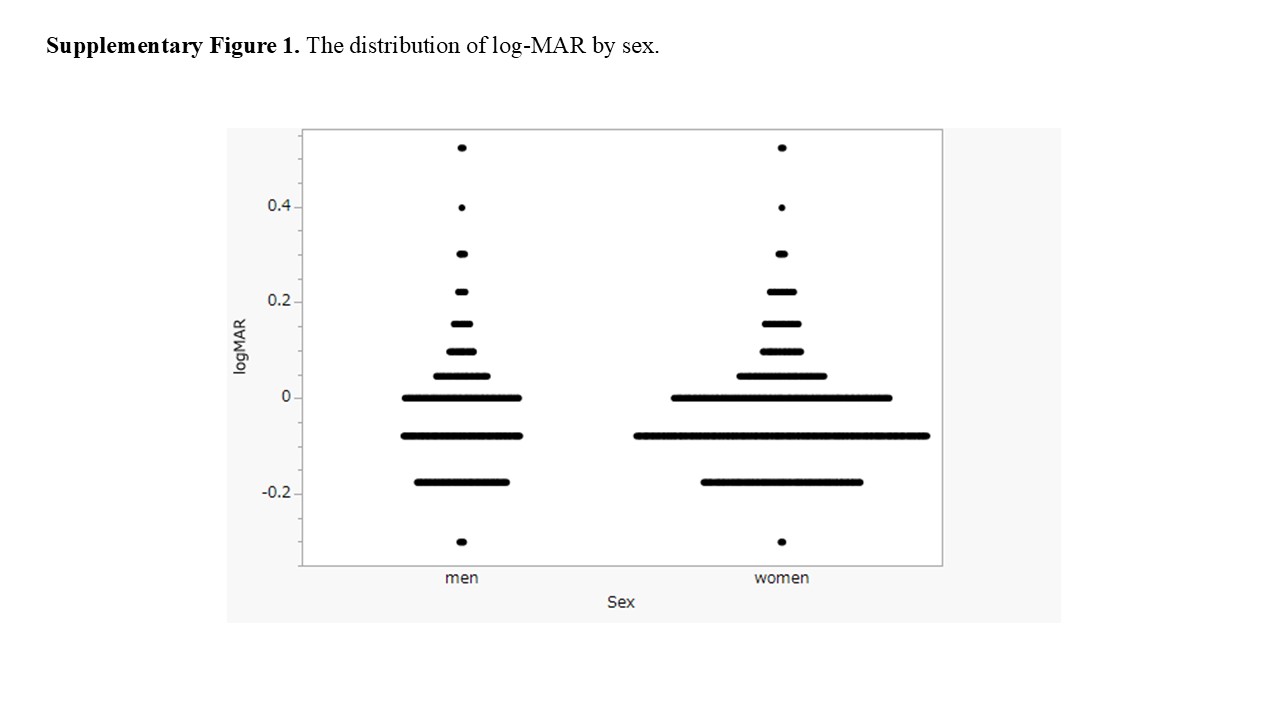

Supplement: Supplementary Figure 1 — shows the distribution of logMAR by sex. [file Image1.jpeg]

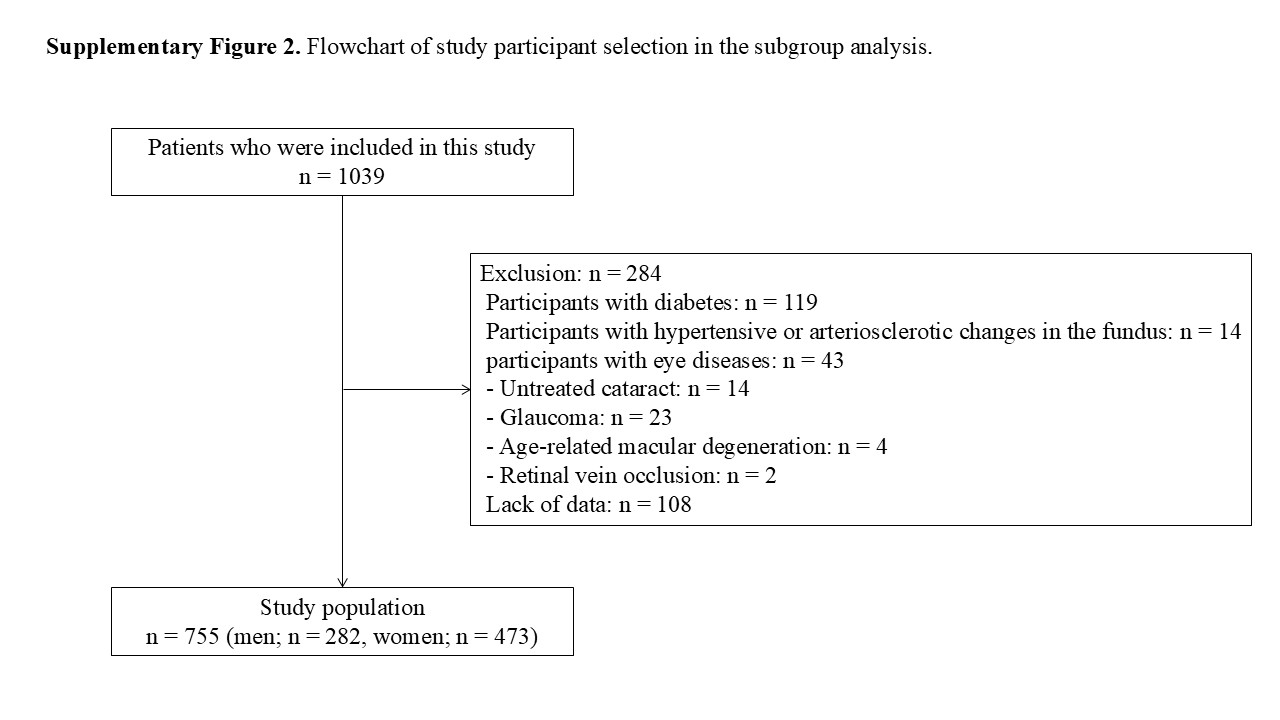

Supplement: Supplementary Figure 2 — shows the flowchart of participant selection for the subgroup analysis. [file Image2.jpeg]
