# Supplementary material for: Association of insulin resistance with visual decline in older individuals without diabetes: a cross-sectional mediation analysis
Source: Front Endocrinol (Lausanne). 2026 Mar 5;17:1758444. doi: 10.3389/fendo.2026.1758444 (PMC12999418; doi:10.3389/fendo.2026.1758444)
Supplement: Supplementary file 3 [file Table1.docx]

**Supplementary Table 1.** Characteristics of study participants in the subgroup analysis.

|  | Total (n=755) | Men (n=282) | Women (n=473) |
| --- | --- | --- | --- |
| Sex (%) | 755 (100) | 282 (37) | 473 (63) |
| Age (years) | 73.9±6.0 | 74.8±6.7 | 73.4±5.5 |
| Body mass index (kg/m^2^) | 23.0±3.1 | 23.4±2.9 | 22.8±3.2 |
| Fat mass (kg) (n=730) | 16.5±5.5 | 15.5±5.4 | 17.0±5.6 |
| Percentage of body fat (%) (n=730) | 29.0±7.5 | 24.3±6.2 | 31.8±6.7 |
| Skeletal muscle mass (kg) (n=730) | 21.3±4.5 | 25.8±3.6 | 18.6±2.1 |
| Skeletal muscle mass index (kg/m^2^) (n=730) | 6.4±1.0 | 7.3±0.8 | 5.9±0.6 |
| Soft lean mass (kg) (n=730) | 37.5±7.1 | 44.7±5.7 | 33.2±3.4 |
| Fat free mass (kg) (n=730) | 39.7±7.4 | 47.2±6.0 | 35.2±3.5 |
| Systolic blood pressure (mmHg) | 138.0±17.5 | 137.6±18.2 | 138.3±17.1 |
| Diastolic blood pressure (mmHg) | 76.7±9.8 | 77.5±10.3 | 76.3±9.4 |
| TG (mg/dL) | 116.2±62.6 | 120.8±62.6 | 113.5±62.6 |
| HDL-C (mg/dL) | 68.9±17.8 | 63.3±17.0 | 72.2±17.5 |
| LDL-C (mg/dL) | 123.7±30.9 | 118.7±28.5 | 126.7±31.9 |
| FIB-4 index | 2.0±0.7 | 2.1±0.8 | 1.9±0.7 |
| HbA1c (%) | 5.7±0.4 | 5.7±0.4 | 5.7±0.4 |
| Fasting plasma glucose (mg/dL) | 101.4±11.0 | 102.4±11.2 | 100.7±10.8 |
| IRI (μU/mL) | 4.7 (3.4-6.7) | 4.5 (3.2-6.5) | 4.8 (3.6-6.9) |
| HOMA-R | 1.2 (0.8-1.7) | 1.1 (0.8-1.7) | 1.2 (0.8-1.7) |
| Free testosterone (pg/mL) | 2.2±2.9 | 5.5±2.1 | 0.2±0.1 |
| Estradiol (pg/mL) (n=754) | 13.3±10.8 | 23.1±9.5 | 7.4±6.5 |
| BCVA | 1.11±0.26 | 1.12±0.28 | 1.11±0.25 |
| LogMAR | -0.079 (-0.079-0.0) | 0.0 (-0.079-0.011) | -0.079 (-0.079-0.0) |
| Current smoker (yes) (%) | 35 (4.4) | 29 (3.6) | 6 (0.8) |
| Past smoker (yes) (%) | 142 (19.2) | 135 (18.1) | 7 (1.1) |
| Non-smoker (yes) (%) | 578 (76.4) | 118 (15.6) | 460 (60.9) |
| Drinking (yes) (%) | 287 (38) | 189 (67) | 98 (21) |
| Academic background (%) | 207 (28) | 88 (32) | 119 (26) |
| Sleep duration (hours) (n=737) | 7.2±1.3 | 7.4±1.3 | 7.0±1.2 |

TG: Triglyceride, HDL-C: High Density Lipoprotein Cholesterol, LDL-C: Low Density Lipoprotein Cholesterol, FIB-4 index: Fibrosis 4 index, IRI: Immunoreactive insulin, HOMA-R: Homeostasis model assessment ratio, BCVA: Best-corrected visual acuity, LogMAR: Logarithmic minimum angle of resolution.

**Supplementary Table 2A.** Relationship between BCVA and HOMA-R.

| HOMA-R | Men (n=297) | | Women (n=500) | | Interaction |
| --- | --- | --- | --- | --- | --- |
|  | β | p | β | p | p |
| Simple regression analysis | -0.033 | 0.5743 | -0.198 | <0.0001 | 0.0039 |
| Multiple regression analysis (Model 1) | -0.001 | 0.9860 | -0.1345 | 0.0026 | 0.0191 |
| Multiple regression analysis (Model 2)  (Men/Women (n=287/485)) | 0.001 | 0.9863 | -0.124 | 0.0063 | 0.0170 |
|  |  |  |  |  |  |
| IRI | Men (n=297) | | Women (n=500) | | Interaction |
|  | β | p | β | p | p |
| Simple regression analysis | -0.038 | 0.5152 | -0.193 | <0.0001 | 0.0068 |
| Multiple regression analysis (Model 1) | -0.017 | 0.7702 | -0.129 | 0.0033 | 0.0423 |
| Multiple regression analysis (Model 2) | -0.016 | 0.7745 | -0.119 | 0.0076 | 0.0345 |
| (Men/Women (n=287/485)) |  |  |  |  |  |

Model 1 was adjusted for age, body mass index (BMI), FIB-4 index, smoking status (current or past smoker), drinking habits, and academic background.

Model 2 was further adjusted for free testosterone, estradiol, and sleep duration.

BCVA: Best-corrected visual acuity, HOMA-R: Homeostasis model assessment ratio, IRI: Immunoreactive insulin.

**Supplementary Table 2B.** Relationship between logMAR and HOMA-R in the subgroup analysis.

| HOMA-R | Men (n=282) | | Women (n=473) | | Interaction |
| --- | --- | --- | --- | --- | --- |
|  | β | p | β | p | p |
| Simple regression analysis | 0.008 | 0.8910 | 0.218 | <0.0001 | 0.0008 |
| Multiple regression analysis (Model 1) | -0.021 | 0.7417 | 0.161 | 0.0005 | 0.0084 |
| Multiple regression analysis (Model 2)  (Men/Women (n=273/463)) | -0.017 | 0.5986 | 0.146 | 0.0019 | 0.0053 |
|  |  |  |  |  |  |
| IRI | Men (n=282) | | Women (n=473) | | Interaction |
|  | β | p | β | p | p |
| Simple regression analysis | 0.008 | 0.8904 | 0.223 | <0.0001 | 0.0006 |
| Multiple regression analysis (Model 1) | -0.008 | 0.8980 | 0.160 | 0.0004 | 0.0110 |
| Multiple regression analysis (Model 2) | -0.003 | 0.9574 | 0.145 | 0.0016 | 0.0066 |
| (Men/Women (n=273/463)) |  |  |  |  |  |

Model 1 was adjusted for age, body mass index (BMI), FIB-4 index, smoking status (current or past smoker), drinking habits, and academic background.

Model 2 was further adjusted for free testosterone, estradiol, and sleep duration.

LogMAR: Logarithmic minimum angle of resolution, HOMA-R: Homeostasis model assessment ratio, IRI: Immunoreactive insulin.

**Supplementary Table 2C.** Relationship between BCVA and HOMA-R in the subgroup analysis.

| HOMA-R | Men (n=282) | | Women (n=473) | | Interaction |
| --- | --- | --- | --- | --- | --- |
|  | β | p | β | p | p |
| Simple regression analysis | -0.032 | 0.5886 | -0.183 | <0.0001 | 0.0112 |
| Multiple regression analysis (Model 1) | 0.003 | 0.9627 | -0.130 | 0.0051 | 0.0418 |
| Multiple regression analysis (Model 2)  (Men/Women (n=273/463)) | 0.002 | 0.9699 | -0.115 | 0.0139 | 0.0300 |
|  |  |  |  |  |  |
| IRI | Men (n=282) | | Women (n=473) | | Interaction |
|  | β | p | β | p | p |
| Simple regression analysis | -0.035 | 0.5566 | -0.184 | <0.0001 | 0.0116 |
| Multiple regression analysis (Model 1) | -0.010 | 0.8636 | -0.126 | 0.0053 | 0.0580 |
| Multiple regression analysis (Model 2) | -0.014 | 0.8186 | -0.112 | 0.0143 | 0.0408 |
| (Men/Women (n=273/463)) |  |  |  |  |  |

Model 1 was adjusted for age, body mass index (BMI), FIB-4 index, smoking status (current or past smoker), drinking habits, and academic background.

Model 2 was further adjusted for free testosterone, estradiol, and sleep duration.

BCVA: Best-corrected visual acuity, HOMA-R: Homeostasis model assessment ratio, IRI: Immunoreactive insulin.

**Supplementary Table 3A.** Total, direct, and indirect effects of BCVA on HOMA-R in various mediating factors.

| Exposure | Mediator | Total effect | p | Natural direct effect | p | Natural indirect effect | p | Proportion mediated (%) | p |
| --- | --- | --- | --- | --- | --- | --- | --- | --- | --- |
| BCVA | Fat mass  Model 1 | -1.395 (-3.089--0.172) | 0.017 | -0.835 (-1.799--0.090) | 0.031 | -0.190 (-0.459-0.043) | 0.112 | 0.136 (-0.075-0.358) | 0.111 |
|  | Model 2 | -1.376 (-2.985--0.140) | 0.015 | -0.825 (-1.687--0.050) | 0.036 | -0.191 (-0.426--0.050) | 0.125 | 0.138 (-0.094-0.402) | 0.122 |
|  | Percentage of body fat  Model 1  Model 2 | -1.572 (-3.516--0.260)  -1.520 (-3.328--0.214) | 0.007  0.015 | -0.822 (-1.810--0.072)  -0.753 (-1.689--0.012) | 0.030  0.045 | -0.230 (-0.528--0.011)  -0.239 (-0.514--0.009) | 0.038  0.035 | 0.146 (0.015-0.358)  0.157 (0.003-0.407) | 0.037  0.048 |
|  | Skeletal muscle mass  Model 1  Model 2 | -1.297 (-2.752--0.173)  -1.183 (-2.554--0.009) | 0.020  0.050 | -1.334 (-2.638--0.297)  -1.245 (-2.408--0.184) | 0.005  0.017 | 0.054 (-0.033-0.168)  0.066 (-0.022-0.193) | 0.246  0.172 | -  - | 0.266  0.222 |
|  | Skeletal muscle mass index  Model 1  Model 2 | -1.220 (-2.508--0.213)  -1.174 (-2.382--0.143) | 0.015  0.017 | -1.173 (-2.253--0.232)  -1.147 (-2.214--0.188) | 0.009  0.014 | -0.019 (-1.160-0.118)  -0.011 (-0.144-0.120) | 0.777  0.894 | -  - | 0.770  0.881 |
|  | Soft lean mass  Model 1  Model 2 | -1.304 (-2.679--0.187)  -1.197 (-2.681-0.003) | 0.018  0.052 | -1.331 (-2.528--0.311)  -1.247 (-2.464--0.145) | 0.006  0.016 | 0.050 (-0.029-0.158)  0.061 (-0.024-0.179) | 0.269  0.215 | -  - | 0.287  0.265 |
|  | Fat free mass  Model 1  Model 2 | -1.313 (-2.719--0.135)  -1.214 (-2.788--0.067) | 0.020  0.037 | -1.328 (-2.596--0.277)  -1.253 (-2.483--0.224) | 0.007  0.011 | 0.050 (-0.031-0.164)  0.060 (-0.028-0.177) | 0.287  0.211 | -  - | 0.307  0.248 |

Model 1 was adjusted for age, FIB-4 index, smoking status (current or past smoker), drinking habits, and academic background.

Model 2 was further adjusted for free testosterone, estradiol, and sleep duration.

BCVA: Best-corrected visual acuity, LogMAR: Logarithmic minimum angle of resolution, HOMA-R: Homeostasis model assessment ratio.

**Supplementary Table 3B.** Total, direct, and indirect effects of logMAR on HOMA-R in various mediating factors in the subgroup analysis.

| Exposure | Mediator | Total effect | p | Natural direct effect | p | Natural indirect effect | p | Proportion mediated (%) | p |
| --- | --- | --- | --- | --- | --- | --- | --- | --- | --- |
| LogMAR | Fat mass  Model 1 | 4.842 (0.252-11.390) | 0.031 | 4.516 (0.250-10.604) | 0.032 | 2.258 (-0.887-7.349) | 0.229 | 0.466 (-0.887-0.880) | 0.214 |
|  | Model 2 | 4.623 (-0.173-11.114) | 0.064 | 4.331 (-0.095-10.427) | 0.058 | 1.998 (-1.290-7.207) | 0.279 | 0.432 (-1.200-1.185) | 0.245 |
|  | Percentage of body fat  Model 1  Model 2 | 5.120 (0.377-11.899)  5.183 (0.035-12.078) | 0.029  0.048 | 4.753 (0.237-11.121)  4.815 (-0.036-11.218) | 0.031  0.063 | 2.584 (-0.626-8.067)  2.557 (-0.557-8.514) | 0.140  0.150 | 0.505 (-0.352-0.940)  0.493 (-0.584-1.105) | 0.125  0.144 |
|  | Skeletal muscle mass  Model 1  Model 2 | 3.471 (0.067-7.367)  3.115 (-0.364-7.382) | 0.044  0.097 | 3.580 (0.203-7.461)  3.267 (-0.179-7.519) | 0.031  0.066 | -0.337 (-1.617-0.812)  -0.481 (-1.842-0.599) | 0.551  0.411 | -  - | 0.587  0.498 |
|  | Skeletal muscle mass index  Model 1  Model 2 | 4.215 (0.276-10.089)  3.629 (-0.118-8.689) | 0.035  0.062 | 4.117 (0.318-9.643)  3.580 (-0.045-8.453) | 0.030  0.057 | 0.581 (-1.157-4.454)  0.294 (-1.433-3.190) | 0.526  0.731 | -  - | 0.511  0.701 |
|  | Soft lean mass  Model 1  Model 2 | 3.541 (0.012-7.692)  3.079 (-0.506-7.425) | 0.047  0.103 | 3.647 (0.171-7.806)  3.223 (-0.317-7.551) | 0.034  0.082 | -0.316 (-1.626-0.917)  -0.440 (-1.964-0.792) | 0.594  0.447 | -  - | 0.641  0.542 |
|  | Fat free mass  Model 1  Model 2 | 3.553 (0.080-7.535)  3.127 (-0.479-7.577) | 0.043  0.123 | 3.664 (0.248-7.634)  3.275 (-0.259-7.604) | 0.034  0.090 | -0.312 (-1.652-0.851)  -0.421 (-1.742-0.920) | 0.597  0.489 | -  - | 0.636  0.602 |

Model 1 was adjusted for age, FIB-4 index, smoking status (current or past smoker), drinking habits, and academic background.

Model 2 was further adjusted for free testosterone, estradiol, and sleep duration.

LogMAR: Logarithmic minimum angle of resolution, HOMA-R: Homeostasis model assessment ratio.

**Supplementary Table 3C.** Total, direct, and indirect effects of BCVA on HOMA-R in various mediating factors in the subgroup analysis.

| Exposure | Mediator | Total effect | p | Natural direct effect | p | Natural indirect effect | p | Proportion mediated (%) | p |
| --- | --- | --- | --- | --- | --- | --- | --- | --- | --- |
| BCVA | Fat mass  Model 1 | -1.217 (-2.988--0.035) | 0.045 | -0.936 (-1.973--0.038) | 0.038 | -0.096 (-0.356-0.141) | 0.421 | 0.079 (-0.439-0.384) | 0.404 |
|  | Model 2 | -1.122 (-2.774-0.065) | 0.068 | -0.877 (-1.909-0.052) | 0.065 | -0.086 (-0.330-0.142) | 0.468 | 0.077 (-0.516-0.476) | 0.450 |
|  | Percentage of body fat  Model 1  Model 2 | -1.395 (-3.026--0.087)  -1.255 (-2.936-0.000) | 0.038  0.050 | -1.041 (-1.918--0.062)  -0.891 (-1.864-0.040) | 0.039  0.063 | -0.115 (-0.384-0.087)  -0.121 (-0.365-0.090) | 0.297  0.279 | 0.082 (-0.189-0.296)  0.096 (-0.320-0.477) | 0.289  0.281 |
|  | Skeletal muscle mass  Model 1  Model 2 | -1.189 (-2.591--0.095)  -1.052 (-2.436-0.117) | 0.032  0.090 | -1.264 (-2.641--0.193)  -1.164 (-2.449--0.014) | 0.015  0.046 | 0.044 (-0.063-0.161)  0.062 (-0.030-0.198) | 0.420  0.223 | -  - | 0.440  0.309 |
|  | Skeletal muscle mass index  Model 1  Model 2 | -1.246 (-2.738--0.106)  -1.123 (-2.388-0.014) | 0.026  0.058 | -1.134 (-2.310--0.128)  -1.068 (-2.074-0.018) | 0.021  0.055 | -0.042 (-0.210-0.096)  -0.020 (-0.153-0.114) | 0.546  0.763 | -  - | 0.536  0.739 |
|  | Soft lean mass  Model 1  Model 2 | -1.193 (-2.592--0.063)  -1.072 (-2.442-0.157) | 0.035  0.091 | -1.264 (-2.595--0.170)  -1.175 (-2.464--0.016) | 0.017  0.044 | 0.043 (-0.054-0.153)  0.060 (-0.030-0.181) | 0.421  0.225 | -  - | 0.450  0.306 |
|  | Fat free mass  Model 1  Model 2 | -1.214 (-2.601--0.048)  -1.065 (-2.593-0.121) | 0.034  0.083 | -1.285 (-2.582--0.142)  -1.165 (-2.580--0.041) | 0.019  0.041 | 0.045 (-0.050-0.163)  0.061 (-0.022-0.184) | 0.383  0.184 | -  - | 0.413  0.263 |

Model 1 was adjusted for age, FIB-4 index, smoking status (current or past smoker), drinking habits, and academic background.

Model 2 was further adjusted for free testosterone, estradiol, and sleep duration.

BCVA: Best-corrected visual acuity, LogMAR: Logarithmic minimum angle of resolution, HOMA-R: Homeostasis model assessment ratio.

**Supplementary Table 4A.** Standardized indirect effects of fat mass and percent of body fat in the mediation analysis of the association between visual function and HOMA-R.

| Exposure | Mediator | Standardized total effect | p | Standardized direct effect | p | Standardized indirect effect | p | Standardized proportion mediated (%) | p |
| --- | --- | --- | --- | --- | --- | --- | --- | --- | --- |
| LogMAR | Fat mass  Model 1 | 0.173 (0.028-0.317) | 0.009 | 0.148 (0.011-0.285) | 0.028 | 0.043 (0.004-0.094) | 0.029 | 0.248 (0.026-0.817) | 0.036 |
|  | Model 2 | 0.162 (0.019-0.306) | 0.024 | 0.137 (0.004-0.274) | 0.040 | 0.042 (0.001-0.090) | 0.041 | 0.261 (-0.032-0.849) | 0.059 |
|  | Percentage of body fat  Model 1  Model 2 | 0.166 (0.027-0.304)  0.165 (0.009-0.291) | 0.010  0.037 | 0.139 (0.005-0.266)  0.137 (-0.012-0.258) | 0.035  0.085 | 0.051 (0.009-0.107)  0.051 (0.007-0.104) | 0.012  0.017 | 0.306 (0.066-1.090)  0.311 (0.007-1.533) | 0.022  0.048 |
| BCVA | Fat mass  Model 1  Model 2 | -0.119 (-0.222--0.015)  -0.107 (-0.218--0.006) | 0.021  0.038 | -0.100 (-0.198--0.003)  -0.087 (-0.198-0.008) | 0.042  0.078 | -0.007 (-0.023-0.006)  -0.008 (-0.023-0.005) | 0.284  0.266 | 0.061 (-0.066-0.431)  0.072 (-0.103-0.529) | 0.293  0.296 |
|  | Percentage of body fat  Model 1  Model 2 | -0.119 (-0.219--0.013)  -0.088 (-0.208--0.002) | 0.028  0.042 | -0.097 (-0.191-0.005)  -0.065 (-0.182-0.015) | 0.066  0.105 | -0.005 (-0.021-0.010)  -0.006 (-0.020-0.011) | 0.405  0.421 | 0.045 (-0.139-0.441)  0.070 (-0.184-0.627) | 0.423  0.441 |

Model 1 was adjusted for age, FIB-4 index, smoking status (current or past smoker), drinking habits, and academic background.

Model 2 was further adjusted for free testosterone, estradiol, and sleep duration.

BCVA: Best-corrected visual acuity, LogMAR: Logarithmic minimum angle of resolution, HOMA-R: Homeostasis model assessment ratio.

**Supplementary Table 4B.** Standardized indirect effects of fat mass and percent of body fat in the mediation analysis of the association between visual function and HOMA-R in the subgroup analysis.

| Exposure | Mediator | Standardized total effect | p | Standardized direct effect | p | Standardized indirect effect | p | Standardized proportion mediated (%) | p |
| --- | --- | --- | --- | --- | --- | --- | --- | --- | --- |
| LogMAR | Fat mass  Model 1 | 0.180 (0.015-0.343) | 0.029 | 0.166 (0.008-0.317) | 0.033 | 0.026 (-0.013-0.076) | 0.227 | 0.145 (-0.221-0.529) | 0.230 |
|  | Model 2 | 0.149 (-0.001-0.310) | 0.053 | 0.136 (-0.004-0.285) | 0.064 | 0.023 (-0.019-0.073) | 0.288 | 0.157 (-0.390-0.724) | 0.289 |
|  | Percentage of body fat  Model 1  Model 2 | 0.166 (0.015-0.314)  0.157 (0.001-0.319) | 0.027  0.048 | 0.149 (0.006-0.292)  0.141 (-0.006-0.295) | 0.036  0.067 | 0.030 (-0.009-0.076)  0.030 (-0.008-0.080) | 0.126  0.138 | 0.178 (-0.102-0.637)  0.188 (-0.206-0.892) | 0.139  0.158 |
| BCVA | Fat mass  Model 1  Model 2 | -0.118 (-0.240--0.008)  -0.109 (-0.242-0.003) | 0.035  0.057 | -0.109 (-0.231--0.003)  -0.100 (-0.226-0.007) | 0.047  0.072 | -0.004 (-0.017-0.010)  -0.004 (-0.017-0.009) | 0.626  0.612 | 0.032 (-0.167-0.276)  0.034 (-0.323-0.406) | 0.629  0.621 |
|  | Percentage of body fat  Model 1  Model 2 | -0.125 (-0.246--0.013)  -0.113 (-0.232-0.004) | 0.030  0.062 | -0.114 (-0.230--0.005)  -0.101 (-0.217-0.010) | 0.046  0.069 | -0.004 (-0.016-0.009)  -0.004 (-0.017-0.009) | 0.593  0.554 | 0.029 (-0.135-0.274)  0.037 (-0.217-0.409) | 0.595  0.568 |

Model 1 was adjusted for age, FIB-4 index, smoking status (current or past smoker), drinking habits, and academic background.

Model 2 was further adjusted for free testosterone, estradiol, and sleep duration.

BCVA: Best-corrected visual acuity, LogMAR: Logarithmic minimum angle of resolution, HOMA-R: Homeostasis model assessment ratio.
